# Risk Factors of High-Grade CIN or Cervix Cancer in Young Women with Abnormal Pap Smear Results: Who Should Be Treated with LEEP (Loop Electrosurgical Excision Procedure)?

**DOI:** 10.3390/jcm14197011

**Published:** 2025-10-03

**Authors:** Hye-Yon Cho

**Affiliations:** Department of Obstetrics and Gynecology, Dongtan Sacred-Heart Hospital, Hallym University Medical Center, Hallym University College of Medicine, Kyeonggido 18450, Republic of Korea; jugilove@gmail.com; Tel.: +82-10-9206-9124; +82-31-8086-2650

**Keywords:** young adults, high-risk HPV, CIN3, CIS, cervix cancer, LEEP, conization

## Abstract

**Objective**: This study aimed to identify risk factors associated with high-grade cervical intraepithelial neoplasia (CIN3+) in young adults with abnormal Pap smears. **Methods**: We performed a retrospective chart review of women ≤30 years who underwent loop electrosurgical excision procedure (LEEP) for abnormal Pap results (atypical squamous cells of undetermined significance [ASCUS] or higher), between 2012 and 2022 at Dongtan Sacred Heart Hospital. Clinical characteristics, including age, HPV infection, prior gynecologic surgery, pelvic inflammatory disease (PID), complete blood count, and Pap smear screening history were collected. Women with CIN3+ based on punch biopsy or LEEP were designated as CIN3+. **Results**: A total of 158 women underwent LEEP. Of these, 61.4% were diagnosed with CIN3+ and 8.2% with invasive cervical cancer. Independent predictors of CIN3+ included age >28 years, smoking, lack of regular Pap screening, and high-risk HPV infection. Subgroup analysis suggested age ≥28 years and neutrophil-to-lymphocyte ratio >2.12 were risk factors for invasive cervical cancer. **Conclusions**: Young Korean women with abnormal Pap smears and risk factors such as older age, smoking, high-risk HPV infection, and irregular screening histories are at increased risk for CIN3+. These findings highlight the importance of timely intervention; however, because our cohort included only women who underwent LEEP, it may represent a higher-risk subset and thus introduce selection bias. Validation in larger multicenter, prospective studies incorporating fertility and recurrence outcomes are needed before definitive recommendations can be made.

## 1. Introduction

This retrospective study aimed to identify risk factors of CIN3+ in Korean women aged ≤30 years presenting with abnormal Pap smear results. Cervical cancer remains a significant public health concern worldwide. Yet, the incidence of cervical intraepithelial neoplasia (CIN) and carcinoma in situ (CIS) has increased in younger age groups, including in Korea [1]. As we know persistent High-risk HPV infection is the leading cause of cervical cancer, HPV vaccination is expected to decrease the incidence of cervical cancer [2]. Despite the introduction of HPV vaccination, coverage among Korean women aged 20–29 remains below 40%, and screening uptake is suboptimal [3]. These gaps underscore the need for data specific to young women in Korea. Recent strategic approaches highlight fertility-preserving and individualized management in early-stage cervical cancer [4].

In Korea, the incidence rate of CIS has increased in all age groups, and specifically, adenocarcinoma in situ has increased in the 20–29 age group [5].

The rising incidence of high-grade CIN and CIS in young adults may be attributed to inadequate participation in regular Pap smear screening [6,7].

However, there have been no data regarding high-risk factors of CIN3/CIS or cancer development in young women. Therefore, in this retrospective study, we aimed to evaluate the risk factors of CIN3+ in young women with abnormal Pap smears.

## 2. Materials and Methods

We retrospectively reviewed the medical records of women under 30 years of age who underwent LEEP for abnormal Pap smear findings between 2013 and 2022 at Hallym University Dongtan Sacred Heart Hospital. A total of 158 patients were included in the study.

Inclusion criteria and indications are as follows: We included women aged ≤ 30 years who underwent LEEP for abnormal cytology (≥ASC-US) at our center. During the study period, LEEP was generally performed according to contemporaneous guideline-concordant indications, including histologically confirmed CIN2+; persistent high-grade cytology (ASC-H/HSIL); or discordant findings with a colposcopic impression highly suspicious for HSIL despite lower-grade cytology. A minority of cases with ASC-US or LSIL proceeded to LEEP when additional risk features were present (e.g., HPV16/18 positivity, persistent abnormalities ≥ 12 months, or high-grade colposcopic impression). We acknowledge that limiting inclusion to patients undergoing LEEP may introduce selection bias, potentially favoring those perceived as being at a higher risk.

Two gynecologic pathologists independently reviewed histopathologic slides from punch biopsy and conization. Both were blinded to patients’ clinical characteristics and outcomes to minimize bias.

The following clinical variables were collected: age, parity, body weight, height, medical history, Pap smear screening history (defined as having undergone screening within the past 3 years), and complete blood count (CBC) results obtained within 1 week before conization. Pap history was verified through medical records when available; when not, it was self-reported at the time of the procedure. Pathologic data included HPV genotype and histopathologic findings from punch biopsy and/or conization specimens.

HPV testing was performed using the Hybrid Capture 2 (HC2) assay (Qiagen, Gaithersburg, MD, USA), which detects the presence of 13 high-risk HPV types as a pooled result but does not provide individual genotype information. Therefore, results were categorized as high-risk HPV positive or negative, without type-specific detail beyond HPV16/18 in cases where additional testing was clinically performed. Longitudinal data on HPV persistence were not consistently available in the medical records, and persistence could not be evaluated in this cohort.

High-grade cervical intraepithelial neoplasia (CIN) was defined as CIN2 or CIN3, based on the pathologic results of either the punch biopsy or the conization specimen. In cases where the two reports differed, the higher-grade lesion was used as the final diagnosis.

Statistical analyses were performed using SPSS software version 27.0 (SPSS Inc., Chicago, IL, USA) and MedCalc version 15.2.2 (MedCalc Software, Ostend, Belgium). Continuous variables with a Gaussian distribution are presented as mean ± standard deviation (SD), and categorical variables are expressed as counts and percentages (n [%]).

Group comparisons were performed using the Pearson chi-squared test or Fisher’s exact test for categorical variables, and the student t-test for continuous variables. A *p*-value < 0.05 was considered statistically significant.

Multivariate analysis was conducted using binary logistic regression to identify independent risk factors. Results are presented as odds ratios (OR) with corresponding 95% confidence intervals (CI). The optimal cutoff value for the neutrophil-to-lymphocyte ratio (NLR) in predicting high-grade CIN was determined using receiver operating characteristic (ROC) curve analysis.

This study was approved by the Institutional Review Board of Hallym University Dongtan Sacred Heart Hospital (IRB No. 2023-02-017-001).

## 3. Results

A total of 158 women aged ≤ 30 years who underwent loop electrosurgical excision procedure (LEEP) for abnormal Pap smear results were included in this study. The mean age was 26.3 ± 3.03 years, and the mean body mass index (BMI) was 22.4 ± 3.96 kg/m^2^. Most participants were nulliparous, with a mean parity of 0.3 ± 0.573. High-risk human papillomavirus (HPV) infection was detected in 87.3% of the cohort, with HPV types 16 or 18 identified in 44.3% of cases (Table 1).

### 3.1. Final Histopathological Analysis Showed

CIN3+ lesions (defined as CIN3 (45), carcinoma in situ [CIS] (38), adenocarcinoma in situ [AIS] (1), or invasive cancer (13)) in 97 patients (61.4%)
Invasive cervical cancer in 13 patients (8.2%)
Lower-grade lesions (CIN1 or CIN2) in 58 patients (36.7%)

### 3.2. Comparison Between CIN3+ and CIN3− Groups

Among the 158 women who underwent LEEP, 97 (61.4%) were diagnosed with CIN3+ lesions, while 61 (38.6%) were classified as CIN3−. Compared to the CIN3− group, women in the CIN3+ group were significantly more likely to:

Be older than 28 years (37.1% vs. 13.1%, *p* = 0.001)
Be current smokers (14.4% vs. 3.3%, *p* = 0.022)
Have never undergone regular Pap screening (70.1% vs. 54.1%, *p* = 0.031)
Test positive for high-risk HPV infection (93.8% vs. 77.1%, *p* = 0.009)

Conversely, the history of prior gynecologic surgery was more prevalent in the CIN3− group (9.9% vs. 2.1%, *p* = 0.038), potentially reflecting more frequent gynecologic surveillance or incidental lesion detection (Table 2).

### 3.3. Multivariate Analysis of CIN3+ Risk Factors

Binary logistic regression identified several independent risk factors significantly associated with CIN3+ lesions:

Age > 28 years: OR 11.165 (95% CI: 3.450–36.132), *p* < 0.001
No history of Pap screening: OR 3.633 (95% CI: 1.451–9.091), *p* = 0.006
High-risk HPV infection: OR 12.512 (95% CI: 2.450–63.909), *p* = 0.002
Current smoking: OR 7.824 (95% CI: 1.467–41.732), *p* = 0.016

HPV 16/18 infection was not significantly associated with CIN3+ in multivariate analysis (OR 1.187; *p* = 0.700) (Table 3).

### 3.4. Predictors of Invasive Cervical Cancer

Of the 97 patients with CIN3+, 13 (13.4%) were diagnosed with invasive cervical cancer. When compared to those with CIN3/CIS/AIS (N = 84), patients with invasive disease were significantly more likely to:

Be ≥ 28 years old (92.3% vs. 44.0%, *p* = 0.001)
Have a neutrophil-to-lymphocyte ratio (NLR) > 2.12 (53.8% vs. 25.0%, *p* = 0.039)

Have single, rather than multiple, HPV infections (7.7% vs. 39.3%, *p* = 0.019) (Table 4).

**Multivariate Analysis of Cervical Cancer Risk Factors**

Independent predictors of invasive cervical cancer in young women included:

Age ≥ 28 years: OR 17.302 (95% CI: 1.736–172.490), *p* = 0.015
NLR > 2.12: OR 5.033 (95% CI: 1.110–22.810), *p* = 0.036

Other variables, including HPV 16/18 infection, a BMI of ≥ 21, and multiple HPV infections, were not statistically significant in the multivariate analysis (Table 5).

## 4. Discussion

In this retrospective study of young women (≤30 years) who underwent LEEP for abnormal Pap smears, we identified several significant risk factors for CIN3+ and invasive cervical cancer. Among the 158 women, 61.4% were ultimately diagnosed with CIN3+ and 8.2% with invasive cervical cancer, highlighting the importance of timely and appropriate intervention even in younger age groups.

While LEEP remains an effective treatment for high-grade cervical lesions, concerns about its impact on fertility and obstetric outcomes must be considered, particularly in young women. Meta-analyses have demonstrated that LEEP is associated with an increased risk of preterm birth, low birthweight, and cervical insufficiency, with risks correlating to cone depth and volume [8,9]. These findings underscore the importance of balancing oncologic safety with reproductive health when recommending excisional treatment in this age group.

Our analysis revealed that age ≥28 years, smoking, high-risk HPV infection, and lack of regular cervical screening were independently associated with the presence of CIN3+. These findings are consistent with earlier studies reporting that persistent high-risk HPV infection and missed screening opportunities are the main contributors to high-grade cervical lesions in younger populations [10].

Notably, older age within the ≤30-year-old group emerged as a strong predictor of CIN3+ and invasive cancer, suggesting that age cutoff points may have clinical utility in risk stratification.

Our findings should also be considered in the context of international guidelines. The 2019 ASCCP risk-based management guidelines recommend surveillance as an option for selected CIN2 cases in young women, particularly when fertility preservation is a priority and close follow-up is feasible [11]. In contrast, our data suggest that certain high-risk features—such as older age within this young cohort, smoking, or irregular screening—are associated with an increased risk of CIN3+ and may justify earlier excisional treatment. Similarly, ESGO/ESTRO/ESP and NICE guidelines emphasize individualized risk stratification, shared decision-making, and counseling about potential obstetric sequelae after excision [12,13]. Thus, while our results align with the principle of individualized management, they highlight that LEEP may be appropriate in specific higher-risk subgroups of young women who might otherwise be considered for surveillance.

Our results suggest that young women with certain high-risk features (e.g., age ≥28, smoking, high-risk HPV, irregular Pap follow-up) may have an increased risk of CIN3+ and could be considered for closer monitoring or early intervention. However, the decision for LEEP in this population must be carefully weighed against potential harm, including cervical insufficiency and adverse obstetric outcomes. Alternative approaches, such as careful observation or ablative therapies, may be appropriate in selected cases.

The association between elevated NLR and invasive disease may reflect both neutrophilia driven by tumor-associated inflammation and relative lymphopenia, indicating impaired anti-tumor immunity [14,15]. In our study, a higher NLR was associated with invasive disease, consistent with meta-analytic evidence supporting its prognostic value in cervical cancer. Clinically, if validated, NLR could assist in triaging young patients for closer surveillance or expedited excision when considered alongside cytology, HPV genotype, and colposcopic findings. However, its utility as a biomarker remains uncertain, as infections, chronic inflammatory conditions, and other non-oncologic factors can influence it. Future studies should investigate the integration of NLR with HPV-based triage strategies to enhance risk stratification in young women with abnormal cytology.

This study adds value to the current body of knowledge by explicitly focusing on young Korean women—a population with relatively low HPV vaccination coverage and unique sociocultural barriers to gynecologic care. Also, we focused on the presence of high-risk HPV and HPV16/18 status; persistence of infection and other oncogenic genotypes were not uniformly captured. This likely attenuates risk discrimination, and future studies incorporating persistence metrics and broader genotyping are warranted.

Nevertheless, our study has several limitations. It was a single-center, retrospective analysis, which may restrict generalizability. Because our cohort included only women who underwent LEEP, it may represent a higher-risk subset, introducing selection bias. The sample size was relatively small, with only 13 cases of invasive cancer, which limited the power of subgroup analyses. Importantly, HPV persistence data were not available, despite persistence being a stronger predictor of progression than single positivity. Moreover, HPV genotyping was primarily restricted to HPV16/18, reducing the granularity of risk stratification. NLR findings should also be interpreted cautiously, as infections, inflammation, or other non-oncologic factors may confound them. Finally, we did not assess recurrence, fertility, or obstetric outcomes, which are central to the management debate in this population.

Future research should prioritize multicenter, prospective studies with larger and more diverse cohorts, standardized HPV genotyping and persistence assessment, and extended follow-up to capture recurrence, fertility, and obstetric outcomes. Such studies are essential to validate our findings and to inform evidence-based recommendations for the management of young women with abnormal cytology. Despite these limitations, our data adds to the growing body of evidence highlighting the unique clinical considerations in very young patients.

## Figures and Tables

**Table 1:** Patients’ characteristics (N = 158).

Characteristics	Mean ± SD or N (%)
Age	26.3 ± 3.03
BMI	22.4 ± 3.96
Parity	0.3 ± 0.573
High-risk HPV infection	138 (87.3)
HPV 16 and/or 18 infections	70 (44.3)
Final pathology	
Negative or chronic cervicitis	3(1.9)
CIN1	10 (6.3)
CIN2	48 (30.4)
CIN3	45 (28.5)
CIS	38 (24.1)
Adenocarcinoma in situ	1 (0.6)
Invasive cervical cancer	13 (8.2)

**Table 2:** Clinical factors comparing women with final diagnosis of CIN3+ and those with CIN3−.

Characteristics	CIN3− (N = 61)	CIN3+ (N = 97)	*p*-Value
Age (years old)			0.001 *
28≤	53 (86.9)	61 (62.9)	
>28	8 (13.1)	36 (37.1)	
BMI (kg/m^2^)	22.6 ± 3.72	22.3 ± 4.12	0.607
No parity	14 (23.0)	24 (24.7)	0.477
Present Smoker			0.022 *
No	59 (96.7)	83 (85.6)	
Yes	2 (3.3)	14 (14.4)	
Prior history of gynecologic surgery			0.038 *
No	55 (90.1)	95 (97.9)	
Yes	6 (9.9)	2 (2.1)	
History of PID			0.198
No	51 (83.6)	74 (76.3)	
Yes	10 (16.4)	23 (23.7)	
Prior PAP			0.031 *
Regular check-up	28 (45.9)	29 (29.9)	
Never done	33 (54.1)	68 (70.1)	
High-risk HPV infection			<0.009 *
Negative	14 (22.9)	6 (6.2)	
Positive	47 (77.1)	91 (93.8)	
HPV 16 or 18 infection			0.079
Negative	39 (63.9)	49 (50.5)	
Positive	22 (36.1)	48 (49.5)	
NLR			0.280
1.6≤	25 (41.0)	34 (35.1)	
>1.6	36 (59.0)	63 (64.9)	

Data are presented as Mean ± SD or N (%). * *p*-Value less than 0.05.

**Table 3:** High-risk factors of CIN3+ in young women.

Risk Factors	OR (95% CI)	*p*-Value
Age (>28 years old)	11.165 (3.450~36.132)	<0.001 *
Prior pap (never done)	3.633 (1.451–9.091)	0.006 *
High-risk HPV infection	12.512 (2.450~63.909)	0.002 *
HPV 16 or 18 infection	1.187 (0.496–2.840)	0.700
Prior history of gynecologic surgery	2.389 (0.294~19.439)	0.416
Smoking	7.824 (1.467~41.732)	0.016 *

* *p*-Value less than 0.05.

**Table 4:** Clinical factors comparing women with final diagnosis of CIN3/CIS/AIS and those with invasive cervical cancer.

Characteristics	CIN3/CIS/AIS (N = 84)	Cervical Cancer (N = 13)	*p*-Value
Age (years old)			0.001 *
28 <	47 (56.0)	1 (7.7)	
≥28	37 (44.0)	12 (92.3)	
BMI (kg/m^2^)	21.9 ± 3.92	24.2 ± 4.99	0.076
No parity	20 (23.8)	4 (30.8)	0.406
Present Smoker			0.385
No	71 (84.5)	12 (93.3)	
Yes	13 (15.5)	1 (6.7)	
Prior PAP			0.414
Regular check-up	26 (31.0)	3 (23.1)	
Never done	58 (69.0)	10 (76.9)	
High-risk HPV infection			0.159
Negative	6 (7.1)	2 (15.4)	
Positive	78 (92.9)	11 (84.6)	
HPV 16 or 18 infection			0.133
Negative	45 (53.6)	4 (30.8)	
Positive	39 (46.4)	9 (69.2)	
HPV multiple infection	33 (39.3)	1 (7.7)	0.019 *
NLR			0.039 *
2.12≤	63 (75.0)	6 (46.2)	
>2.12	21 (25.0)	7 (53.8)	

* Data are presented as Mean ± SD or N (%).

**Table 5:** High-risk factors of cervical cancer in young women.

Risk Factors	OR (95% CI)	*p*-Value
Age (≥8 years old)	17.302 (1.736~172.490)	0.015 *
BMI (≥21)	4.412 (0.869~22.397)	0.073
HPV 16 or 18 infection	2.779 (0.613~12.599)	0.185
multiple HPV infection	0.134 (0.014~1.304)	0.083
NLR (>2.12)	5.033 (1.110~22.810)	0.036 *

* *p*-Value less than 0.05.

## Data Availability

Data is unavailable due to privacy or ethical restrictions.
